# *Veillonella rogosae* sp. nov., an anaerobic, Gram-negative coccus isolated from dental plaque

**DOI:** 10.1099/ijs.0.65093-0

**Published:** 2008-03

**Authors:** Nausheen Arif, Thuy Do, Roy Byun, Evelyn Sheehy, Douglas Clark, Steven C. Gilbert, David Beighton

**Affiliations:** 1 Department of Microbiology, The Henry Wellcome Laboratories for Microbiology and Salivary Research, King's College London Dental Institute, Floor 17, Guys Tower, London Bridge, London SE1 9RT, UK; 2 Institute of Dental Research, Westmead Centre for Oral Health and Westmead Millennium Institute, Westmead Hospital, Wentworthville, NSW 2145, Australia

## Abstract

Strains of a novel anaerobic, Gram-negative coccus were isolated from the supra-gingival plaque of children. Independent strains from each of six subjects were shown, at a phenotypic level and based on 16S rRNA gene sequencing, to be members of the genus *Veillonella*. Analysis revealed that the six strains shared 99.7 % similarity in their 16S rRNA gene sequences and 99.0 % similarity in their *rpoB* gene sequences. The six novel strains formed a distinct group and could be clearly separated from recognized species of the genus *Veillonella* of human or animal origin. The novel strains exhibited 98 and 91 % similarity to partial 16S rRNA and *rpoB* gene sequences of *Veillonella parvula* ATCC 10790^T^, the most closely related member of the genus. The six novel strains could be differentiated from recognized species of the genus *Veillonella* based on partial 16S rRNA and *rpoB* gene sequencing. The six novel strains are thus considered to represent a single novel species of the genus *Veillonella*, for which the name *Veillonella rogosae* sp. nov. is proposed. The type strain is CF100^T^ (=CCUG 54233^T^=DSM 18960^T^).

At the time of writing, the genus *Veillonella* comprised the following recognized species: *Veillonella parvula*, *V. atypica*, *V. dispar*, *V. criceti*, *V. ratti*, *V. rodentium*, *V. caviae, V. montpellierensis* and *V. denticariosi* (Mays *et al.*, 1982; Rogosa, 1984; Jumas-Bilak *et al.*, 2004; Byun *et al.*, 2007). Members of the genus are non-fermentative, anaerobic, Gram-negative, small cocci isolated from the oral cavity and intestinal tract of humans and animals that gain energy from the utilization of short-chain organic acids (Delwiche *et al.*, 1985). Species of the genus isolated from humans are *V. parvula*, *V. atypica*, *V. dispar*, *V. montpellierensis* and *V. denticariosi*, although a strain described as a member of the *V. ratti* –*V. criceti* group has been found in a semen sample from a patient attending for treatment of infertility at a urology unit (Marchandin *et al.*, 2005). The above species may be associated with monomicrobial infections in humans (Marchandin *et al*., 2001).

Identification of taxa to the genus *Veillonella* is quite straightforward but the identification of members of the genus to the species level is difficult owing to the lack of discriminating phenotypic tests (Kolenbrander & Moore, 1992). Analysis of 16S rRNA gene sequences permits the identification of isolates as members of the genus *Veillonella*. However, very low levels of 16S rRNA gene sequence dissimilarity are found within the genus and, although *V. atypica*, *V*. *montpellierensis* and the species isolated from rodents are identifiable by this means, discrimination between *V. dispar*, *V. parvula* and *V. denticariosi* is problematic (Jumas-Bilak *et al.*, 2004; Byun *et al.*, 2007). Additionally, there have been frequent reports of the occurrence of intra-chromosomal heterogeneity between 16S rRNA genes in human isolates of *Veillonella* species, further complicating the identification of isolates on the basis of 16S rRNA gene sequencing (Marchandin *et al.*, 2003). In order to overcome the limitations associated with using 16S rRNA gene sequences to identify *Veillonella* species, comparisons of partial *dnaK*, *rpoB* and *gyrB* gene sequences have been proposed as a reliable method to identify members of the genus. All recognized members of the genus can be discriminated by using *dnaK* gene sequence comparison and, in particular, *V. parvula* and *V. dispar* have been reliably identified in this way (Jumas-Bilak *et al.*, 2004). In a more recent report, the ability of *dnaK* gene sequencing to differentiate between *Veillonella* species, including *V. denticariosi*, was confirmed, and comparisons of *rpoB* and *gyrB* gene sequences were also shown to enable the identification of species within the genus, with the *rpoB* gene being the more discriminatory (R. Byun, personal communication).

As part of a large study investigating the genotypic diversity of *Veillonella* species present in the dental plaque of caries-free children and in the infected dentine of carious lesions, we have frequently isolated strains that appeared based on 16S rRNA gene sequencing to be distinct from all recognized species of the genus. The aim of the present study was to determine the taxonomic status of these isolates by using a polyphasic approach.

Occlusal or buccal surface plaque was collected from nine caries-free children. The children or their carers gave consent to the collection of the samples and the study was approved by the local ethics committee (ref. 02/11/10). *Veillonella*-like isolates were recovered on agar consisting of (per litre deionized water) 5 g Bacto tryptone (Difco), 5 g Bacto yeast extract (Difco), 0.75 g sodium thioglycolate (Sigma), 0.002 g Bacto basic fuchsin (Difco), 21 ml 60 % sodium lactate (Sigma) and 15 g Bacto agar (Difco). The pH was adjusted to 7.5 prior to autoclaving; vancomycin (7.5 μg ml^−1^; Sigma) was added following autoclaving (Rogosa, 1956). Inoculated plates were incubated in an atmosphere consisting initially of 90 % (v/v) nitrogen, 5 % (v/v) hydrogen and 5 % (v/v) carbon dioxide at 37 °C for 4 days. Isolates were presumptively identified as members of the genus *Veillonella* on the basis of their ability to grow on the selective isolation medium and their typical colonial appearance: colonies were 2–4 mm in diameter, regular and slightly domed in shape with an entire edge.

To confirm the identity of the isolates as belonging to the genus *Veillonella*, genomic DNA was obtained from each isolate by suspending colonies in deionized water and heating the cells to 95 °C for 10 min. The 16S rRNA gene was amplified by using the primers 27f and 1492r (Lane, 1991) and sequencing was performed by using the 27f primer in conjunction with Big Dye Ready Reaction Termination Mix (ABI) to obtain short 16S rRNA gene sequences of 450–500 nt. These sequences were submitted to the Ribosomal Database Project (http://rdp.cme.msu.edu/) via the Sequence Match routine. The majority (>99 %) of isolates were identified as members of the genus *Veillonella*.

The partial 16S rRNA gene sequences (450–500 nt) of all the new isolates were aligned by using clustal w (Thompson *et al.*, 1994) in BioEdit (http://www.mbio.ncsu.edu/BioEdit/bioedit.html) with partial 16S rRNA gene sequences of *V. caviae* DSM 20738^T^, *V. rodentium* ATCC 17743^T^, *V. montpellierensis* ADV 281.99^T^, *V. parvula* ATCC 10790^T^, *V. dispar* ATCC 17748^T^, *V. atypica* ATCC 17744^T^, *V. ratti* ATCC 17746^T^, *V. criceti* ATCC 17747^T^ and *V. denticariosi* CIP 109448^T^. Phylogenetic relationships between taxa were analysed by using mega 3.1 (Kumar *et al.*, 2004). Distances were calculated according to the Kimura two-parameter model and clustering was based on the neighbour-joining method of Saitou and Nei (1987) by using bootstrap values based on 1000 replicates. From these analyses, a group of isolates was identified that appeared to represent a novel taxon that was distinct from all other clinical isolates and from other type and reference strains investigated (data not shown).

In order to understand the phylogenetic position of these isolates more clearly, more complete 16S rRNA gene sequences (1355 nt) were obtained from single strains (designated CF100^T^, CF05, CF30, CF84, CF88 and CF24), each isolated from a different, unrelated subject. The 16S rRNA gene sequences of these six strains, and also of *V. parvula* ATCC 10790^T^, exhibited a high level of sequence similarity with many cloned 16S rRNA gene sequences from taxa isolated from the human mouth or gut (data not shown). Alignment of the longer 16S rRNA gene sequences revealed similarity of >99.3 % (mean 99.7 %) among the six novel strains, demonstrating that they belonged to the same species. They showed the highest 16S rRNA gene sequences similarity to *V. parvula* ATCC 10790^T^ (98 %). Phylogenetic relationships between the new isolates and recognized species of the genus *Veillonella* were investigated as described above with *Dialister pneumosintes* ATCC 33048^T^ as the outgroup organism. The resulting neighbour-joining tree is shown in Fig. 1. When the 16S rRNA gene sequence data were analysed by using the minimum-evolution method in mega 3.1 (Kumar *et al.*, 2004), the resultant tree had the same appearance with the novel strains on a distinct branch separated from other *Veillonella* species with a bootstrap value of 32 %. When the interior branch test of phylogeny (Kumar *et al.*, 2004) was employed to test the significance of the branch containing the six novel strains, the confidence probability obtained with the neighbour-joining and minimum-evolution methods was 84 and 88 %, respectively, indicating that discrimination between *V. parvula* and the novel strains was approaching significance. However, 16S rRNA gene sequence analysis does not reliably distinguish between *Veillonella* species (Jumas-Bilak *et al.*, 2004; Byun *et al.*, 2007).

To overcome the above difficulty, comparisons of *dnaK*, *gyrB* and *rpoB* gene sequences have been used to differentiate between *Veillonella* species, including *V. denticariosi* (Jumas-Bilak *et al.*, 2004; Byun *et al.*, 2007). We used this approach in this study to determine the position of the six novel strains more accurately. No amplicons were produced when we used the primers previously described for the *dnaK* gene (Jumas-Bilak *et al.*, 2004). We therefore used primers for the *rpoB* gene (Veill-rpoBF, GTAACAAAGGTGTCGTTTCTCG; Veill-rpoBR, GCACCRTCAAATACAGGTGTAGC) and were successful in obtaining and sequencing amplicons from all the strains investigated. The sequences (608 nt) were aligned, as described above, with the *rpoB* gene sequences of *V. parvula* strains ATCC 10790^T^, ATCC 17745, RBV162, RBV167, RBV180, RBV182, RBV175, RBV30, RBV123, RBV156, RBV173, RBV184, RBV54, RBV186, RBV188, RBV35, RBV136, RBV192, RBV183, RBV159, RBV146, RBV78, RBV179 and RBV185, *V. atypica* ATCC 17744^T^, *V. dispar* ATCC 17748^T^, *V. denticariosi* CIP 109448^T^, *V. caviae* DSM 20738^T^, *V. criceti* ATCC 17747^T^, *V. ratti* ATCC 17746^T^, *V. rodentium* ATCC 17743^T^ and *V. montpellierensis* ADV 281.99^T^. *Pseudoalteromonas atlantica* T6c was used as the outgroup organism. The six novel strains formed a distinct taxon with robust bootstrap support (99 %) in the *rpoB* gene tree (Fig. 2). When the interior branch test of phylogeny was employed to test the significance of the branch containing the novel strains, the confidence probability obtained with the neighbour-joining and minimum-evolution methods was 99 %, indicating robust discrimination between *V. parvula* and the six novel strains. Mean *rpoB* gene sequence similarity among the six novel strains was 99.0 % and was 98.4 % among *V. parvula* strains, indicating homogeneity of the two taxa. Mean *rpoB* gene sequence similarity between the novel strains and strains belonging to *V. parvula*, the most closely related species, was 91 %, indicating that strains CF100^T^, CF05, CF30, CF84, CF88 and CF24 represent a novel species. We suggest that as 16S rRNA gene sequence comparisons are not suitable for differentiating between all members of the genus *Veillonella*, comparisons based on *rpoB* gene sequences represent a valid method for species characterization. This approach is validated given that *V. dispar*, *V. parvula* and *V. denticariosi* cannot be separated satisfactorily by using 16S rRNA gene sequence comparisons but can be defined clearly when *rpoB* gene sequence data are considered (Fig. 2).

As strains CF100^T^, CF05, CF30, CF84, CF88 and CF24 formed a phylogenetically distinct group based on *rpoB* gene sequencing, with robust bootstrap support, separate from *V. parvula* and all other recognized *Veillonella* species, we suggest that they represent a novel species of the genus *Veillonella*. We undertook a more complete phenotypic analysis of these strains.

Strains CF100^T^, CF05, CF30, CF84, CF88 and CF24 were tested, at least twice, by using the rapid ID 32A and API 20A identification kits (bioMérieux). Cells were small, Gram-negative coccoids, mainly appearing singly but with some short chains visible. Spores were not formed. No sugars were fermented in either of the identification kits. All strains were catalase-negative and did not hydrolyse aesculin, but one strain (CF84) weakly hydrolysed arginine. All isolates reduced nitrate. All were positive for pyroglutamic acid arylamidase and alkaline phosphatase was produced weakly by three of the six strains; all other test results were negative. No carbohydrates were fermented to acids in either test system. All strains fermented lactate, but not glucose, in peptone/yeast extract broth to produce major amounts of both propionic and acetic acids as the only acid end products when culture supernatants were analysed by using a GLC methodology (Delwiche *et al.*, 1985). None of the six strains was positive for any of the following enzyme activities (Beighton *et al.*, 1991) as measured by using 4-methyl umbelliferyl-linked fluorogenic substrates: *α*-galactosidase, *β*-galactosidase, *α*-glucosidase, *β*-glucosidase, *β*-*N*-acetylgalactosaminidase, *α*-arabinosidase, *β*-*N*-acetylglucosaminidase, sialidase, *α*-fucosidase or *β*-fucosidase. The strains did not exhibit cytochrome oxidase activity. As with the recently described species *V*. *montpellierensis* (Jumas-Bilak *et al.*, 2004), and other species of the genus (Kolenbrander & Moore, 1992), phenotypic characteristics were not sufficient to differentiate the novel isolates from other members of the genus.

## Description of *Veillonella rogosae* sp. nov.

*Veillonella rogosae* (ro.go.sae. N.L. masc. gen. n. *rogosae* of Rogosa, named in honour of the late American microbiologist Morrison Rogosa, for his outstanding contributions to microbiology and to the taxonomy of the genus *Veillonella*).

Cells are Gram-negative, non-motile, non-sporulating coccoids (0.3–0.5 μm in diameter) that occur singly and in short chains. Colonies on *Veillonella* agar (Rogosa, 1956) are 2–4 mm in diameter and domed with an entire edge. Strictly anaerobic. Able to reduce nitrate, but unable to hydrolyse aesculin or arginine. Oxidase-negative. Unable to produce acids from carbohydrates and does not exhibit extracellular glycosidic enzyme activities. Exhibits pyroglutamic acid arylamidase activity and variable alkaline phosphatase activity. Major acid end products are acetic and propionic acids. Can be differentiated from other species of the genus *Veillonella* based on partial 16S rRNA and *rpoB* gene sequencing.

The type strain, CF100^T^ (=CCUG 54233^T^=DSM 18960^T^), was isolated from supra-gingival plaque of a child. Strains CF05, CF30, CF84, CF88 and CF24, isolated from similar sources, are also included in the species.

## Figures and Tables

**Fig. 1. f1:**
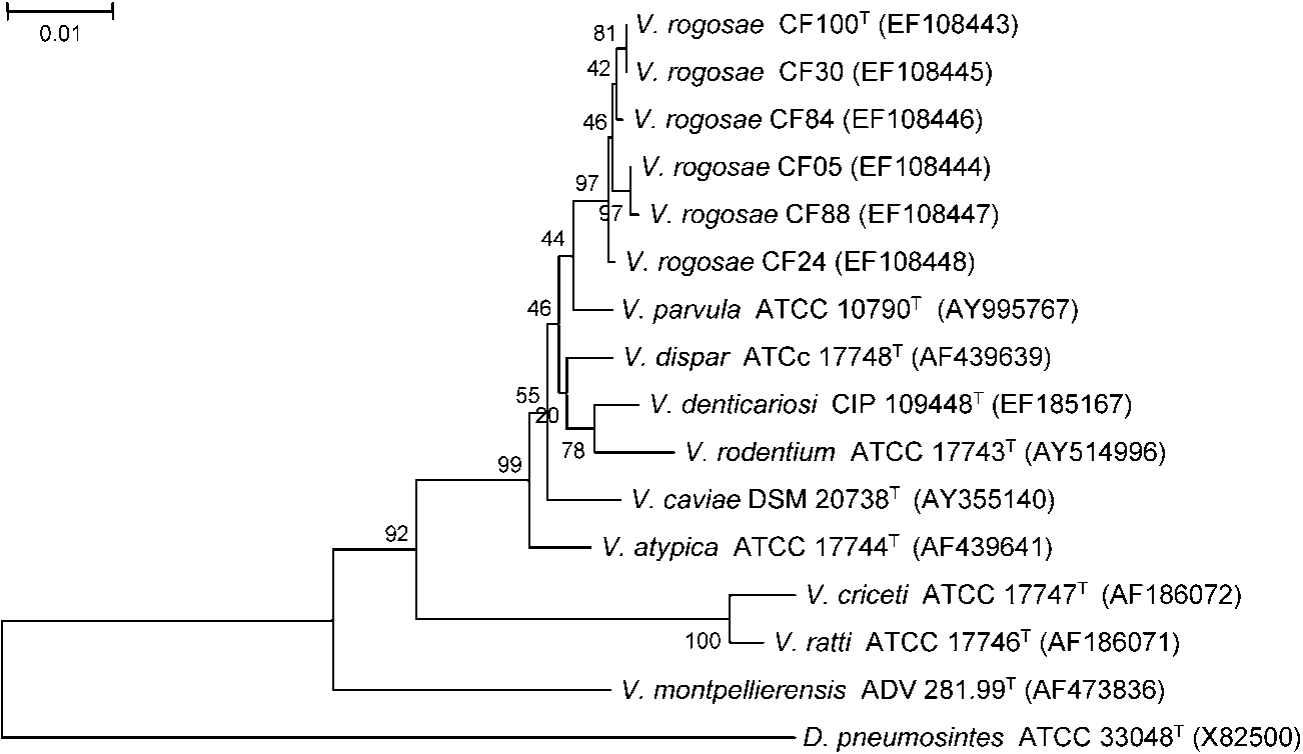
Neighbour-joining tree based on nearly complete 16S rRNA gene sequences (1309 nt), showing relationships between strains CF100^T^, CF05, CF30, CF84, CF88 and CF24 and the type strains of recognized members of the genus *Veillonella*. *Dialister pneumosintes* ATCC 33048^T^ was used as the outgroup organism. Accession numbers for 16S rRNA gene sequences are given for each strain. Bootstrap values are indicated at corresponding nodes. Bar, 0.01 substitutions per site.

**Fig. 2. f2:**
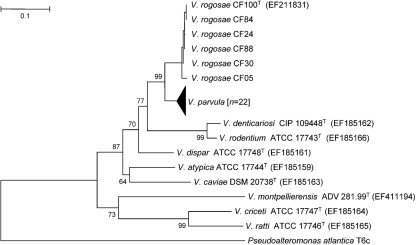
Neighbour-joining tree based on partial *rpoB* gene sequences (608 nt), showing relationships between strains CF100^T^, CF05, CF30, CF84, CF88 and CF24 and recognized members of the genus *Veillonella*. *Pseudoalteromonas atlantica* T6c (gi:109698613 : 682317–686345) was used as the outgroup organism. The strains of *V. parvula* are not listed but were *V. parvula* ATCC 10790^T^ (EF185158), *V. parvula* ATCC 17745 (EF185160) and an additional 22 *rpoB* gene sequences with accession numbers EU077574–EU077595. Bootstrap values are indicated at corresponding nodes. Bar, 0.1 substitutions per site.
